# The role of lncRNA-mediated ceRNA regulatory networks in liver fibrosis

**DOI:** 10.1016/j.ncrna.2024.01.001

**Published:** 2024-01-09

**Authors:** Jianhao Jiang, Ilgiz Gareev, Tatiana Ilyasova, Alina Shumadalova, Weijie Du, Baofeng Yang

**Affiliations:** a Department of Pharmacology (The State-Province Key Laboratories of Biomedicine Pharmaceutics of China, Key Laboratory of Cardiovascular Research, Ministry of Education), College of Pharmacy, 150067, Harbin Medical University, Harbin, China; b Translational Medicine Research and Cooperation Center of Northern China, Heilongjiang Academy of Medical Sciences, Harbin 150081, China; c Department of Internal Diseases, Bashkir State Medical University, Ufa, Republic of Bashkortostan, 3 Lenin Street, 450008, Russia; d Department of General Chemistry, Bashkir State Medical University, Ufa, Republic of Bashkortostan, 3 Lenin Street, 450008, Russia; e Central Research Laboratory, Bashkir State Medical University, Ufa, Republic of Bashkortostan, 3 Lenin Street, 450008, Russia

**Keywords:** lncRNAs, miRNA, ceRNAs, Liver fibrosis, Physiological processes, Early disease diagnosis, Therapeutic strategies

## Abstract

In the dynamic realm of molecular biology and biomedical research, the significance of long non-coding RNAs (lncRNAs) acting as competing endogenous RNAs (ceRNAs) continues to grow, encompassing a broad spectrum of both physiological and pathological conditions. Particularly noteworthy is their pivotal role in the intricate series of events leading to the development of hepatic fibrosis, where hepatic stellate cells (HSCs) play a central role. Recent strides in scientific exploration have unveiled the intricate involvement of lncRNAs as ceRNAs in orchestrating the activation of HSCs. This not only deepens our comprehension of the functioning of proteins, DNA, and the extensive array of coding and noncoding RNAs but also sheds light on the intricate molecular interactions among these molecules. Furthermore, the well-established ceRNA networks, involving classical interactions between lncRNAs, microRNAs (miRNAs), and messenger RNAs (mRNAs), are not mere bystanders; they actively participate in instigating and advancing liver fibrosis. This underscores the pressing need for additional thorough research to fully grasp the potential of ceRNA. The unyielding pursuit of knowledge in this field remains a potent driving force with the capacity to enhance the quality of life for numerous individuals grappling with such diseases. It holds the promise of ushering in a new era of precision medicine, signifying a relentless dedication to unraveling the intricacies of molecular interactions that could pave the way for transformative advancements in the diagnosis and treatment of hepatic fibrosis.

## 1 Introduction

**Fig. 1:**
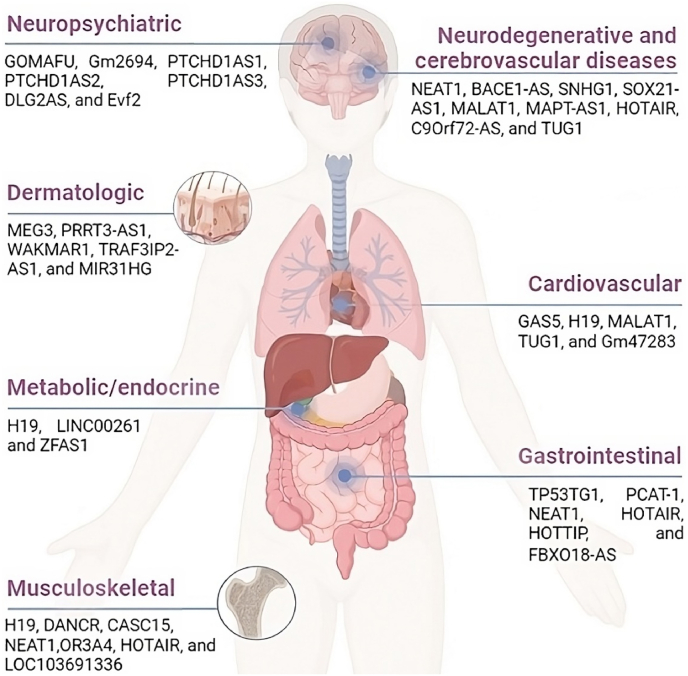
List of the most studied long non-coding RNAs (lncRNAs) in recent years in the conditions grouped by organ and body system.

Liver fibrosis is a pathological condition characterized by the excessive accumulation of extracellular matrix (ECM) in the liver. It primarily results from the prolonged activation of tissue repair mechanisms triggered by recurring liver tissue injuries [1]. The principal contributors to ECM production in the liver are hepatic stellate cells (HSCs) [2,3]. These cells are in the space of Disse, nestled between sinusoidal endothelial cells and hepatic epithelial cells, where they establish intimate connections with both cell types. A unique feature of HSCs is the presence of specialized organelles called lipid droplets, which store hepatic retinoids. The depletion of these lipid droplets may play a role in the development of hepatic diseases [4,5]. A growing body of evidence underscores the significant influence of HSCs on the differentiation, proliferation, and morphogenesis of other cell types within the liver during its development and regenerative processes [6,7]. HSC activation, often referred to as *trans*-differentiation, serves as the primary source of myofibroblasts, which are responsible for secreting matrix proteins and represent the central driving force behind liver fibrosis [8]. This intricate molecular pathway significantly impacts various facets of cell functionality, including cell division, activation, and specialization. HSCs activation can be induced either directly or indirectly, through paracrine signals originating from injured epithelial cells, the fibrotic microenvironment, immune system, systemic metabolic imbalances, enteric dysbiosis, and the presence of hepatitis viral products [9,10]. Previous experimental data suggests that multiple signaling pathways, such as hedgehog (HH), Fas/Fas Ligand (FasL), Notch, p53, and transforming growth factor beta 1 (TGF-β1)/Smad3, can either impede or facilitate the progression of liver fibrosis by influencing the behavior of HSCs [11,16]. Similarly, microRNAs (miRNAs) are recognized for their ability to either promote or alleviate liver fibrosis by affecting these pathways [[12], [13], [14], [15], [16], [17], [18]]. Long non-coding RNAs (lncRNAs) are RNA molecules arbitrarily defined as those longer than 200 nucleotides, primarily for practical reasons related to RNA purification protocols that typically exclude smaller RNAs [19]. These lncRNAs serve as critical intermediaries in transmitting information from higher-order chromosomal interactions to modifications in chromatin structure. They have the potential to orchestrate the organization of chromatin domains, facilitating the long-distance activation of specific genes [20]. Moreover, it's a recurring theme observed in various model systems that lncRNAs form intricate networks of ribonucleoprotein (RNP) complexes, collaborating with numerous chromatin regulators to direct their enzymatic activities to precise locations within the genome. In a broader conceptual sense, lncRNAs represent a versatile class of genes that participate in various biological functions, acting as signals, decoys, guides, and scaffolds [21,22]. In the context of competing endogenous RNAs (ceRNAs), lncRNAs contribute to extensive regulatory networks spanning the transcriptome, substantially expanding the functional genetic information encoded in the human genome. This diversified group of molecules plays a pivotal role in various pathological conditions [[23], [24], [25], [26], [27], [28]] (Fig. 1).

**Fig. 2:**
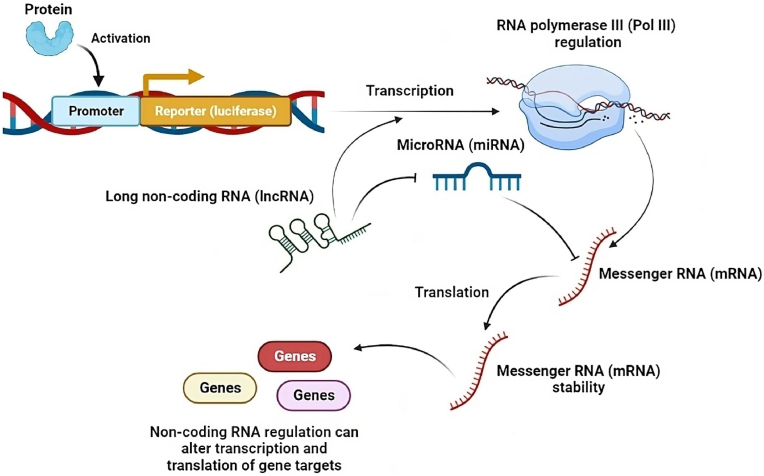
Schematic illustration of long non-coding RNA (lncRNA)-microRNA (miRNA)-messenger RNA (mRNA) interaction.

Crucially, mRNAs are collectively and competitively regulated by both lncRNAs and mRNAs, illustrating a well-established ceRNAs mechanism (Fig. 2).

This mechanism demonstrates how lncRNAs, acting as ceRNAs, can either block or activate mRNAs, ultimately influencing protein production. Recent scientific evidence has firmly established that lncRNAs, when functioning as ceRNAs networks, play a pivotal role in the initiation and progression of hepatocellular carcinoma (HCC), offering potential new strategies for treating this condition [29]. However, the biological and molecular mechanisms underlying the role of lncRNAs in liver fibrosis remain incompletely understood. Therefore, we have synthesized a comprehensive overview of the functional networks and regulatory mechanisms involving lncRNAs as ceRNAs in the context of liver fibrosis that will provide a valuable novel therapeutic approach.

## 2 LncRNAs and their competitive endogenous RNA networks in liver fibrosis

Although liver fibrosis is considered reversible, the resulting structural damage can lead to irreversible cirrhosis. As there is a growing need for diagnostic and prognostic biomarkers for liver fibrosis, lncRNAs have emerged as potential candidates due to their involvement in various diseases through interaction with mRNAs and miRNAs. One study based on the ceRNA hypothesis used data from Gene Expression Omnibus (GEO) datasets to construct a tripartite global network including lncRNA-miRNA-mRNA interactions [30]. The network included 220 lncRNAs, 24 miRNAs, 164 mRNAs, and 1149 interactions. To understand the functions of liver fibrosis -related genes, Gene Ontology (GO) and Kyoto Encyclopedia of Genes and Genomes (KEGG) pathway analyzes were performed, which revealed significant enrichment in terms of cytokine and collagen regulation, as well as TGF-β and toll-like receptors (TLRs) signaling [31]. Notably, TGF-β signaling has been identified as a potential therapeutic target in liver fibrosis, particularly in the activation of HSCs [32]. Concentrated nodes characterized by high connectivity were identified as key elements of the network, with four lncRNAs (NONMMUT036242, XR_877072, XR_378619, and XR_378418) highlighted as potential biomarkers for the diagnosis and treatment of liver fibrosis [33,34]. Although several lncRNAs have been reported to be associated with liver fibrosis and HSC activation, the functional characterization of these key lncRNAs is still in the early stages. To infer their potential functions, ceRNA theory has been applied to study related miRNAs and mRNAs with annotated functions. Key lncRNA-miRNA-mRNA network provided detailed insight into how these lncRNAs interact with competing mRNAs [35]. For example, lncRNA XR_877072, a relatively understudied lncRNA, exhibited interactions with multiple mRNAs, including CD74, discoidin domain receptor 2 (DDR2), and tissue inhibitor of metalloproteinase 2 (TIMP2), all of which are closely associated with liver fibrosis [36,37]. In addition, XR_877072 directly interacted with important miRNAs such as miR-378a-3p and miR-212-3p, both of which are recognized as important factors in the development of liver fibrosis. For example, miR-378a-3p is associated with decreased fibrotic gene expression in liver fibrosis models, while overexpression of miR-212-3p induces HSC marker expression through TGF-β signaling [38,39].

**Fig. 3:**
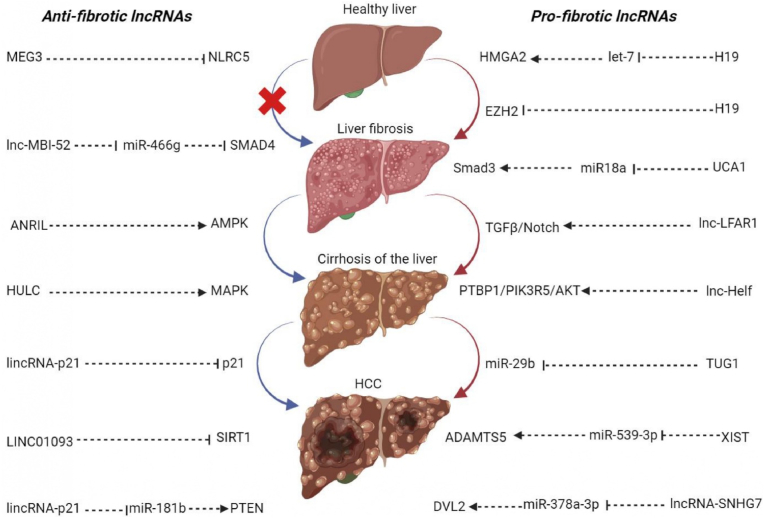
The role of long non-coding RNAs (lncRNAs) in the progression of liver fibrosis. Liver fibrosis occurs when scar tissue replaces damaged cells. Over time, the accumulation of scars deforms the liver, disrupts its blood supply, and can lead to serious consequences: from cirrhosis to hepatocellular carcinoma (HCC). In this context, lncRNAs play an important role not only in the development of liver fibrosis but also in its progression, dividing them into two large groups as anti-fibrotic and pro-fibrotic lncRNAs.

Another study underlines the previously unexplored role of a specific lncRNA, known as lincRNA-p21, in liver fibrosis [40]. Liver fibrosis, characterized by the accumulation of ECM, is a significant medical concern. The research observed a substantial reduction in lincRNA-p21 expression in both mouse liver fibrosis models and human cirrhotic livers, prompting further investigation. To investigate the impact of lincRNA-p21, the study conducted experiments both in vitro and in vivo. Overexpressing lincRNA-p21 in vitro led to the suppression of HSC activation, a critical player in liver fibrosis. Introducing lincRNA-p21 via lentivirus reduced the severity of liver fibrosis in mice. Notably, lincRNA-p21 was found to reverse the activation of HSCs, guiding them back to their quiescent state. An intriguing discovery was the positive correlation between lincRNA-p21 and p21, a well-known cell cycle regulator. This relationship indicated that the overexpression of lincRNA-p21 triggered an increase in both mRNA and protein levels of p21 [40]. This, in turn, inhibited cell-cycle progression and proliferation of primary HSCs. Such insights into the molecular mechanisms involved in liver fibrosis are crucial for developing potential therapeutic strategies. In a clinical context, the study also evaluated serum lincRNA-p21 levels in patients with liver cirrhosis. It revealed significantly lower levels of lincRNA-p21 in these patients, particularly in cases of decompensated cirrhosis. This clinical correlation underscores the relevance of lincRNA-p21 as a mediator of HSC activation and suggests its potential as a novel therapeutic target for liver fibrosis. Overall, this research sheds light on the intricate molecular mechanisms involved in liver fibrosis, providing a promising avenue for future therapeutic interventions [41]. The study of lincRNA-p21 and its role in liver fibrosis opens up new possibilities for the development of treatments that could significantly impact the lives of patients suffering from this condition. Table 1, Table 2, and Fig. 3 show the impact of lncRNA-miRNA-mRNA interaction in promote or the reversal liver fibrosis [[42], [43], [44], [45], [46], [47], [48], [49], [50], [51], [52], [53]].

**Table 1:** Long non-coding RNAs (lncRNAs) act as endogenous RNAs (ceRNAs) that promote liver fibrosis.

LncRNA	Regulation of lncRNA	Study model	miRNA target	Gene target	Function	Reference
ATB	Up	In vitro (human tissue and LX-2 cells)	miR-200a	β-catenin	Facilitate HSC activation and collagen Ι production	[42]
PVT1	Up	In vitro (primary HSCs) and in vivo (male C57BL/6J mice)	miR-152	PTCHl and HH	Stimulate HSC activation and contribute to the development of HH and EMT	[43]
NEAT1	Up	Ex vivo (patients' serum), in vitro (AML-12 cells) and in vivo (male C57BL/6J mice)	miR-129-5p	SOCS2	Stimulate the development of liver fibrosis in alcoholic steatohepatitis	[44]
NEAT1	Up	In vitro (mouse hepatic stellate cell lines (HSCs)) and in vivo (male C57BL/6J mice)	miR-148a-3p and miR-22-3p	Cyth3	Closely related to the progression of liver fibrosis	[45]
HOTAIR	Up	In vitro (AML-12 and RAW264.7 cell line) and in vivo (male C57BL/6J mice)	miR-148b	DNMT1/MEG3/p53	Promotes cell proliferation and activation	[46]
HOTAIR	Up	In vitro (human tissue and primary HSCs) and in vivo (male C57BL/6J mice)	miR-29b	PTEN	Stimulate HSC growth, cell cycle progression, and activation. Activates α-SMA and type I collagen	[47]
NEAT1	Up	In vitro (primary HSCs) and in vivo (male C57BL/6J mice)	miR-122	KLF6	Promotes HSCs proliferation and activation	[48]

**Abbreviations:** ATB, lncRNA activated by transforming growth factor-β; PVT1, Plasmacytoma variant translocation 1; HOTAIR, HOX antisense intergenic RNA; NEAT1, Nuclear paraspeckle assembly transcript 1; HSCs, Hematopoietic stem cells; PTCHl, Phosphatidyl choline; HH, Hedgehog; EMT, Epithelial–mesenchymal transition; SOCS2, Suppressor of cytokine signaling 2; Cyth3, Cytohesin 3; DNMT1, DNA (cytosine-5)-methyltransferase 1; MEG3, Maternally expressed 3; PTEN, Phosphatase and tensin homolog; KLF6, Kruppel-like factor 6.

**Table 2:** Long non-coding RNAs (lncRNAs) act as endogenous RNAs (ceRNAs) that the reversal of liver fibrosis.

LncRNA	Regulation of lncRNA	Study model	miRNA target	Gene target	Function	Reference
MEG3	Down	In vitro (LX2 and primary HSCs) and in vivo (CCl_4_ mice)	miR-212	SMO	Suppresses of liver fibrosis via EMT, with a reduction in α-SMA and type I collagen, and inhibits activation of HSCs	[49]
GAS5	Down	In vitro (LX2 and primary HSCs) and in vivo (male Sprague-Dawley rats)	miR-23a	PTEN/PI3K/Akt /mTOR/Snail	Inhibits liver fibrosis progression	[50]
GAS5	Down	In vitro (human tissue and primary HSCs) and in vivo (male C57BL/6J mice)	miR-222	p27	Inhibits the activation and proliferation of HSCs	[51]
Gm5091	Down	In vivo (male C57BL/6J mice)	miR-27b, miR-23b, and miR-24	IL-1β, α-SMA and Desmin	Inhibits progression of alcoholic liver fibrosis and suppresses HSC activation	[52]
GAS5	Down	In vitro (LX2 cells)	miR-433-3p	NF-κB and TLR10	Inhibitory effect on HSC activation	[53]

**Abbreviations:** MEG3, Maternally expressed 3; GAS5, Growth arrest special 5; HSCs, Hematopoietic stem cells; SMO, Smoothened, frizzled class receptor; PTEN, Phosphatase and tensin homolog; PI3K, Phosphoinositide 3-kinases; mTOR, Mammalian target of rapamycin; IL-1β, Interleukin 1 beta; α-SMA, Alpha-smooth muscle actin; NF-κB, Nuclear factor kappa B; TLR10, Toll Like Receptor 10; EMT, Epithelial–mesenchymal transition.

## 3 LncRNA-ATB

Fu et al. conducted a study to investigate the involvement and molecular mechanisms of the lncRNA-ATB/miR-200a axis in the context of liver fibrosis associated with Hepatitis C virus (HCV) infection. Given the serious health implications of HCV-induced liver fibrosis, comprehending the underlying regulatory processes is crucial for potential therapeutic interventions. The primary aim of the research was to clarify how lncRNA-ATB/miR-200a influences the activation of HSCs and the progression of liver fibrosis in HCV patients. The study employed a dual-luciferase reporter assay to predict and confirm binding sites between lncRNA-ATB/miR-200a and β-catenin. It was established that lncRNA-ATB contains common binding sites for both miR-200a and β-catenin. The results indicated upregulation of lncRNA-ATB in fibrotic liver tissues, activating LX-2 cells treated with conditioned media from cells expressing HCV core protein. Importantly, the study observed a correlation between decreased miR-200a expression, increased β-catenin expression, and liver tissues from HCV-related liver fibrosis patients with activated HSCs. This suggests the involvement of the lncRNA-ATB/miR-200a/β-catenin regulatory axis in liver fibrosis development in HCV patients. Lastly, the research demonstrated that knocking down lncRNA-ATB led to reduced β-catenin expression by upregulating endogenous miR-200a, resulting in the suppression of LX-2 cell activation. In summary, this study provides insights into the regulatory network encompassing lncRNA-ATB, miR-200a, and β-catenin in the context of liver fibrosis associated with HCV. The findings suggest that targeting lncRNA-ATB could be a potential therapeutic approach for treating liver fibrosis in HCV patients. This knowledge enhances our understanding of the molecular mechanisms behind HCV-induced liver fibrosis and opens avenues for future treatment strategies.

## 4 LncRNA PVT1

In their investigation, Zheng et al. delved into the role of the lncRNA known as plasmacytoma variant translocation 1 (PVT1) in the context of liver fibrosis, with a specific focus on HSCs [43]. The study underscores the significance of the EMT process in HSC activation, a pivotal event in the development of liver fibrosis. The HH pathway, known to be crucial for the EMT process, connects it to HSC activation. While PVT1 has been previously linked to various human cancers, its role in liver fibrosis has not been elucidated. The study revealed upregulation of PVT1 in fibrotic liver tissues and its activation of HSC. In vivo experiments demonstrated that PVT1 depletion led to reduced collagen deposition, indicating its involvement in fibrosis development. In vitro experiments showed that lowering PVT1 levels inhibited HSC activation, including reduced proliferation, diminished expression of α-smooth muscle actin (α-SMA) - a marker of activated HSCs - and type I collagen. The study further explored the mechanism through which PVT1 influences HSC activation. Results indicated that PVT1 knockdown inhibited EMT and the HH pathway. Notably, Patched1 (PTCH1), a negative regulator of the HH pathway, was upregulated by PVT1 knockdown. The demethylation of PTCH1 induced by miR-152 was identified as the factor responsible for the effects of PVT1 knockdown on PTCH1 expression. Critically, the inhibition of miR-152 reversed the impact of PVT1 knockdown on HSC activation. The study also confirmed the direct interaction between miR-152 and PVT1 through luciferase reporter assays and pull-down assays. Thus, the research indicates that PVT1 epigenetically suppresses PTCH1 expression by competitively binding miR-152, subsequently promoting the EMT process in the context of liver fibrosis [43].

## 5 LncRNA H19

Two separate yet interconnected studies have elucidated the roles of methyl-CpG binding protein 2 (MeCP2) and the lncRNA H19 in the context of liver fibrosis [44,45]. While conducted independently, these studies collectively provide a comprehensive understanding of the molecular mechanisms underlying diverse aspects of liver fibrosis. In the first study, the focus was on MeCP2 and its involvement in liver fibrosis using a rat model treated with CCl4 to induce fibrosis. The study revealed significant downregulation of H19 in HSCs and fibrotic liver tissues, while MeCP2 and insulin-like growth factor receptor 1 (IGF1R) exhibited an opposite pattern, suggesting their potential roles in fibrogenesis. MeCP2 silencing demonstrated a pronounced inhibitory effect on HSC proliferation, implicating MeCP2 in HSC activation and proliferation, crucial processes in liver fibrosis. Furthermore, MeCP2 knockdown led to increased H19 expression in activated HSCs, revealing a negative regulatory relationship between MeCP2 and H19. The study also explored H19's impact on IGF1R expression, indicating H19's role in regulating IGF1R. Overexpression of H19 was found to inhibit TGF-β1-induced HSC proliferation, suggesting therapeutic potential by targeting MeCP2 and its downstream effectors, including H19 and IGF1R, to mitigate liver fibrosis. The second study delved into the specific role of H19 in cholestatic liver fibrosis, examining its influence in the context of disrupted bile acid homeostasis induced by bile duct ligation (BDL). The study revealed that overexpression of H19 RNA in the liver increased liver fibrosis in BDL mice, accompanied by elevated serum markers of liver damage and altered bile acid levels. The study identified significant changes in the expression of genes related to fibrosis, inflammation, and biliary hyperplasia in H19-BDL mice, along with an enrichment of specific immune cell populations in the liver and spleen, indicating H19's role in modulating immune responses. Mechanistically, the study demonstrated that H19 RNA influenced the expression of zinc finger E-box binding homeobox 1 (ZEB1), epithelial cell adhesion molecule (EpCAM), and SRY (sex-determining region Y)-box 9, suggesting a complex role for H19 in cholestatic liver fibrosis through the ZEB1/EpCAM signaling pathway. These findings underscore the potential of therapeutic strategies targeting H19 to mitigate fibrosis progression. Together, these studies offer valuable insights into the molecular mechanisms involving MeCP2 and H19 in diverse aspects of liver fibrosis. The collective results suggest that these molecules, along with their downstream effectors such as IGF1R and ZEB1, play crucial roles in liver fibrosis and may serve as promising targets for therapeutic interventions. Understanding the intricate interaction of MeCP2 and H19 in both general liver fibrosis and cholestatic liver fibrosis contributes to a broader comprehension of the disease and its potential treatment avenues.

## 6 LncRNA HOTAIR

In an investigation into liver fibrosis, one study explored the impact of HOTAIR, a long intergenic noncoding RNA, on HSCs. The study revealed a substantial increase in HOTAIR expression in both in vivo and in vitro HSCs during the progression of liver fibrosis. Elevated HOTAIR levels were closely linked to HSC activation, as evidenced by heightened α-SMA and type I collagen expression. Knocking down HOTAIR had a suppressive effect on HSC activation, resulting in reduced α-SMA and type I collagen levels and mitigating fibrogenic processes. Additionally, HOTAIR knockdown inhibited HSC proliferation and cell cycle progression, underscoring its role in promoting fibrotic processes, particularly through the regulation of HSC proliferation. Another study uncovered a key mechanistic insight into the influence of HOTAIR on the phosphatase and tensin homolog (PTEN) gene. HOTAIR knockdown led to an increase in PTEN, associated with the loss of DNA methylation. This epigenetic regulation was linked to the effects of miR-29b, which controls PTEN methylation. HOTAIR was identified as a target of miR-29b and acted as a sponge for this miRNA, facilitating the downregulation of PTEN expression. Interestingly, increased HOTAIR expression was also observed in hepatocytes during liver fibrosis. Loss of HOTAIR in hepatocytes resulted in increased PTEN expression and decreased type I collagen, indicating the involvement of HOTAIR in hepatocyte-mediated fibrotic processes. HOTAIR expression was significantly elevated in mouse models of liver fibrosis, human fibrotic livers, and TGF-β1-stimulated HSCs. The study demonstrated that heightened HOTAIR expression in LX-2 cells promoted cell proliferation and activation. Notably, HOTAIR acted as a molecular sponge for miR-148b, playing a critical role in regulating the DNA cytosine 5-methyltransferase 1 (DNMT1)/maternal expression 3 (MEG3)/p53 pathways in HSCs, emphasizing its influence on the epigenetic landscape within these cells. Interestingly, HOTAIR was found to increase the occupancy of the repressive complex 2 (PRC2) and repressive histone marks H3K27me3, particularly in the MEG3 promoter region, indicating its involvement in the epigenetic regulation of fibrogenic processes. Additionally, HOTAIR forms an RNA/DNA hybrid and recruits PRC2 to the MEG3 promoter, highlighting its role in the epigenetic modulation of gene expression in the context of liver fibrosis.

## 7 LncRNA NEAT1

Yu et al. conducted a study investigating the role of NEAT1 in primary mouse HSCs and a carbon tetrachloride (CCl4)-induced mouse liver fibrosis model. The research demonstrated a significant upregulation of NEAT1 expression in mice with CCl4-induced liver fibrosis and in activated HSCs, suggesting a potential involvement of NEAT1 in liver fibrosis progression. Loss of NEAT1 exhibited a pronounced inhibitory effect on liver fibrosis both in vivo and in vitro. Conversely, overexpression of NEAT1 accelerated HSC activation, leading to increased cell proliferation and collagen expression. NEAT1's impact on HSC activation was mediated through the miR-122/Kruppel-like factor 6 (KLF6) axis. The study revealed that the effects of NEAT1 on HSC activation were significantly mitigated by miR-122 mimics or KLF6 knockdown. Intriguingly, both NEAT1 and KLF6 were identified as targets of miR-122, underscoring the complexity of the regulatory network. The study unveiled a direct interaction between miR-122 and NEAT1, indicating that NEAT1 acts as a sponge for miR-122, resulting in the downregulation of this miRNA. Consequently, this downregulation contributes to the activation of HSCs and the progression of liver fibrosis. The impact of the research extended to human fibrotic liver samples, revealing a positive correlation between elevated NEAT1 levels and markers of liver fibrosis, underscoring the clinical significance of these findings. This study unravels a novel signaling cascade involving NEAT1, miR-122, and KLF6, providing profound insights into the molecular mechanisms underlying liver fibrosis. Targeting NEAT1 and its associated miR-122-KLF6 axis may potentially offer innovative therapeutic strategies for liver fibrosis, holding promise for more effective treatments in the future.

## 8 LncRNA GAS5

Li et al. conducted a study on autophagy-related genes (ATGs), specifically focusing on their potential role in addressing liver fibrosis [49]. Their findings revealed that ATGs exerted selective cytotoxicity on platelet-derived growth factor-BB (PDGF-BB)-activated HSCs, inducing cell cycle arrest at the G0/G1 phase. This inhibitory effect was specific to activated HSCs and did not impact normal human hepatocytes. The mechanism involved the modulation of cyclin-dependent kinase (CDK) 4/6 activity, leading to reduced expression of cyclin D1 and CDK4/6, primarily in early G1 phase. Additionally, ATG increased p27Kip1 expression and its association with CDK2, with this effect being significantly diminished when p27Kip1 was suppressed. ATG also interfered with PDGF-BB-induced Akt phosphorylation, influencing the transcription factor Forkhead box O 3a (FOXO3a) and ultimately inhibiting HSC proliferation. In another study, a different researcher explored the role of the lncRNA growth arrest-specific transcript 5 (GAS5) in the regulation of liver fibrosis [50]. GAS5 was found to be downregulated in fibrotic liver samples and activated HSCs. Overexpression of GAS5 significantly suppressed HSC activation and collagen accumulation. A key mechanism involved GAS5 acting as a competing ceRNA for miR-222, leading to the upregulation of p27 protein. MiR-222 inhibits GAS5 expression, and GAS5, in turn, represses miR-222. Through direct binding to miR-222, GAS5 establishes a ceRNA interaction that increases p27 protein levels, effectively inhibiting HSC activation and proliferation. In summary, both ATG and GAS5 present promising approaches to inhibit HSC proliferation in the context of liver fibrosis. ATG selectively targets activated HSCs while sparing healthy hepatocytes, offering a unique therapeutic intervention strategy. On the other hand, GAS5, through its ceRNA role for miR-222, forms a regulatory circuit that ultimately increases p27 levels, suppressing HSC activation and proliferation [[51], [52], [53]].

## 9 Future perspectives

HSCs play a key role in various liver diseases, including liver fibrosis, which, if left untreated or improperly treated, can progress to cirrhosis and even liver cancer. MiRNAs are well known for their vital functions in liver fibrosis, and lncRNAs have also received recognition for their role. For example, the PCA3 lncRNA test has received approval for clinical use, illustrating the potential of lncRNAs as diagnostic and therapeutic candidates. Some lncRNAs, such as HOTAIR, PVT1, lncRNA-ATB, H19, and NEAT1, are upregulated and enhance liver fibrosis, while others, such as lincRNA-p21, GAS5, H19, and Gm5091, are downregulated, promoting the reversal of liver fibrosis [54]. However, compared to protein coding sequences and undiscovered functional lncRNAs, the identified lncRNAs represent only a fraction of what may exist, with most of their functional roles remaining undiscovered. This highlights the need for further study of lncRNAs acting as competing ceRNAs in the initiation and progression of liver fibrosis. The pathophysiology of liver fibrosis is regulated by networks including miRNAs and signaling pathways such as miR-126/(NF-ĸB and Wnt inhibitory factor 1 (WIF1)), miR-200c/(FOG2, epidermal growth factor (EGF), phosphoinositide 3 - kinases (PI3Ks) and AKT) and miR-31/(Smad3, TGF-β and hypoxia-inducible factor-1 (HIF-1)), among others [17,18,24,31,55]. LncRNAs, as hypothetical ceRNAs, influence mRNA expression by competitively interacting with microRNAs. Recent studies have mechanistically explained the occurrence and progression of liver fibrosis through networks of long noncoding ceRNAs can function to enhance HF (EHlncRNAs) and RHlncRNAs. The interaction of these lncRNAs with miRNAs provides insight into the regulation of liver fibrosis. For example, HOTAIR in combination with miR-29b modulates PTEN expression or, alternatively, in tandem with DNMT1 and MEG3 affects p53. PVT1 acts through miR-152 and PTCH1/HH, LncRNA-ATB through miR-200a and β-catenin, H19 through ZEB1 and EpCAM, and NEAT1 through miR-122 and KLF6, to name a few. These interactions, both theoretical and mechanistic, shed light on how lncRNAs play a role in promoting or inhibiting HSC activation, EMT or PTEN expression. Although the concept of lncRNA ceRNA networks is relatively young, it is experimentally elucidating the processes underlying liver fibrosis and opening up potential therapeutic opportunities. Beyond the individual interactions between lncRNAs and miRNAs, there is a broader network of ceRNAs that plays a universal and critical regulatory role in liver fibrosis [56]. LncRNAs exhibit diverse functions as ceRNAs, not limited to their interactions with miRNAs but extending to influence mRNA networks. This highlights the need to pay more attention to lncRNAs [47,48]. Moreover, miRNAs that share common targets with ceRNAs and lncRNAs show promise to inhibit or even reverse liver fibrosis. Regulation of EHlncRNAs is particularly important for the treatment of liver fibrosis. LncRNAs play different roles in different types of liver fibrosis. For example, H19 levels are increased in gastric cancer and biliary cirrhosis, but decreased in liver fibrosis [44,45]. HOTAIR in the context of liver fibrosis may either enhance liver fibrosis by binding to miR-29b and reducing PTEN or promote liver fibrosis by interacting with miR-148b and activating DNMT1/MEG3/p53 [[56], [57], [58]]. Similarly, p21 can improve liver fibrosis by interacting with miR-181b and up-regulating PTEN or can reverse liver fibrosis by binding to miR-17-5p and suppressing the Wnt/β-catenin pathway [49,50]. This cross-regulation of lncRNAs helps shed light on the multifaceted nature of diseases, including liver fibrosis, and supports the idea of targeting multiple factors simultaneously.

## 10 Conclusion

In conclusion, lncRNAs represent a complex and relatively uncharted realm within the context of liver fibrosis. Their diverse roles, whether as promoters or inhibitors of liver fibrosis, position them as promising candidates for both diagnosis and potential therapeutic targets. Furthermore, the extensive competitive ceRNA networks highlight the importance of adopting a comprehensive perspective when studying liver fibrosis. Although this field is still in its early stages, it holds significant promise for unveiling the intricate mechanisms that underlie liver fibrosis and for pioneering innovative therapeutic approaches. As research in this area progresses, we anticipate a deeper understanding and the development of more effective strategies for managing liver fibrosis.

## Funding

This work was supported by the 10.13039/501100001809National Natural Science Foundation of China (82273928, U21A20339) and by the Bashkir State Medical University Strategic Academic Leadership Program (PRIORITY-2030).

## Patient consent for publication

Not applicable.

## Ethics approval and consent to participate

Not applicable.

## Availability of data and materials

Not applicable.

## Publisher's note

All claims expressed in this article are solely those of the authors and do not necessarily represent those of their affiliated organizations, or those of the publisher, the editors and the reviewers. Any product that may be evaluated in this article, or claim that may be made by its manufacturer, is not guaranteed or endorsed by the publisher.

## CRediT authorship contribution statement

**Jianhao Jiang:** Writing – review & editing, Writing – original draft. **Ilgiz Gareev:** Writing – review & editing, Visualization, Conceptualization. **Tatiana Ilyasova:** Formal analysis. **Alina Shumadalova:** Data curation. **Weijie Du:** Writing – review & editing, Project administration, Funding acquisition, Conceptualization. **Baofeng Yang:** Supervision, Project administration, Funding acquisition.

## Declaration of competing interest

The authors declare that the research was conducted in the absence of any commercial or financial relationships that could be construed as a potential conflict of interest.
